# Behavioral observation of prosocial behavior and social initiative is related to preschoolers’ psychopathological symptoms

**DOI:** 10.1371/journal.pone.0225274

**Published:** 2019-11-21

**Authors:** Laura Huber, Maria Plötner, Julian Schmitz

**Affiliations:** 1 Department of Clinical Child and Adolescent Psychology, Institute of Psychology, University of Leipzig, Leipzig, Saxony, Germany; 2 Leipzig Research Centre for Early Childhood Development, University of Leipzig, Leipzig, Saxony, Germany; Institute of Physiology and Basic Medicine, RUSSIAN FEDERATION

## Abstract

Current research on preschool-age psychopathology suggests specific impairments in the two domains of social competence–prosocial behavior and social initiative–in children with externalizing and internalizing symptoms. While behavioral observation methods have been largely neglected in the past, they may extend the predominating questionnaire-based assessment as they allow for a precise and objective assessment of children’s social behavior. In this study, we aimed to investigate whether prosocial behavior and social initiative measured in a limited resource task are related to externalizing and internalizing symptoms in a preschool-age community sample (*N* = 117, *M* = 4.67 years, *SD* = 2.75 months, females = 57). Externalizing and internalizing symptoms were rated by teachers (*n* = 109) and parents (*n* = 77) using the Strengths and Difficulties Questionnaire and by children using the Berkeley Puppet Interview (*n* = 97). Reduced prosocial actions were related to children’s higher ratings of externalizing symptoms, while reduced social initiative actions were related to parents’ higher ratings of internalizing symptoms. Prosocial behavior in the behavioral task was a marginally significant positive predictor of internalizing symptoms from children’s perspective. These results highlight the value of behavioral observation measures and contribute to our understanding of interpersonal deficits already related to psychopathology at preschool age.

## Introduction

Acquiring social competence is seen as a major developmental task and a central component of healthy functioning as early as preschool age [1, 2] and it can be defined as the ability to appropriately balance one’s own needs in conjunction with those of others [3, 4]. Recent research has demonstrated that social competence has positive effects on a variety of domains in children’s lives, such as academic achievement, quality of peer relations, conflict management skills, and life satisfaction [2, 5–7]. These effects have also been found longitudinally, demonstrating the positive effect of early social competence in preschool on social competence and mental health in later schooling and adolescence [8].

Rubin and Rose-Krasnor’s [3] definition of social competence allows for operationalization on a concrete behavioral level. It implies that socially-competent children exhibit other-oriented competence, so-called *prosocial behavior* [3,4, 9]. In standardized behavioral observation tasks, developmental research has shown that preschool-age children robustly show various forms of prosocial behavior such as helping, sharing, informing or comforting others [10–12]. The definition by Rubin and Rose-Krasnor [3] also implies that socially-competent children show self-oriented competence–so-called *social initiative–*for example, social participation, leadership behavior, setting limits to others, verbalizing own needs, and sociability [3, 4, 9, 13, 14]. Developmental research has demonstrated that preschool-age children also show this part of social competence; for example, by ensuring their own needs in a socially-adaptive manner [15, 16]. Even though prosocial behavior and social initiative are both considered social competence skills, it is important to note that empirical data suggests that they are independent from each other [17].

### Social competence and mental health in children

Several theoretical and empirical models–such as developmental task theory [18], resilience theory [19], and prevention science [20]–assume that the acquisition of social competence fosters healthy functioning. In addition, the tripartite model of social competence by Perren and Malti predicts differential effects of self- and other-oriented social competence on children’s health and well-being [4, 21]. The beneficial interpersonal skills of socially-competent children have been highlighted by Green and Rechis [6]; for example, in terms of conflict management and turn-taking skills, knowledge how to share resources equally and the ability to display one’s feelings and adopt the perspective of others. In addition, common models about developing and maintaining mental disorders suggest that social skill deficits are related to mental health problems. For example, in their cognitive model of social phobia, Clark and Wells [22] describe that social skill deficits such as anxiety-related safety behaviors (e.g. avoidance of self-disclosure or gaze-avoidance) are likely to make other people perceive patients with social phobia as less friendly and warm, and consequently also react with less friendly behavior. Thereby, a negative interaction pattern may evolve, which contributes to maintaining the social phobia. In theories of social skills deficits associated with depression (see [23] for an overview), authors point to the negative interactional effects of social skills deficits; for example, in terms of reduced smiling or eye contact. These behaviors lead to reduced positive reinforcement by the social environment, which in turn fosters depression. Furthermore, studies on the trajectory of externalizing symptoms underline the role of reduced social skills especially in terms of prosocial behavior in developing and maintaining externalizing disorders such as attention deficit hyperactivity disorder (ADHD; [24–26]). For example, in children with ADHD, the core symptoms of the disorder–such as inattention and hyperactivity or impulsivity–already make successful peer interactions difficult [25, 27]. Once they have exhibited social skill deficits, children with ADHD often experience peer rejection [27], which in turn may foster the aggravation of related symptoms such as aggressive behavior. Thus, social competence plays a central role in theories of healthy development in children as well as prominent theories on mental disorders in children and adults.

Regarding the specific relationship between the two dimensions of social competence [3] and manifest mental disorders, previous studies have found that externalizing symptoms–for example, characterized by conduct problems, antisocial behavior, hyperactivity, or attention problems [28, 29]–are negatively related to prosocial behavior [4, 30–32]. Social initiative has not yet been investigated to a major extent, although children with externalizing symptoms have been found to display higher levels of aggression and assertiveness [4, 24, 33]. Thus, children with externalizing problems seem to approach others to an overly strong extent and may not be able to balance their own desire in conjunction with the need of others. This suggests that externalizing symptoms are related to rather elevated levels of social initiative. In sum, the more externalizing symptoms that children exhibit, the less that they engage in helping, sharing, and comforting others and the more that they potentially approach others to ensure their own rights and desires.

By contrast, internalizing symptoms–for example, characterized by depression, anxiety, withdrawal, and physiological suffering [28, 29]–have been found to be both negatively [17, 34] and positively [35, 36] related to prosocial behavior. Thus, the more internalizing symptoms that children exhibit, the less *or* more that they potentially engage in helping, sharing, or comforting others. Regarding social initiative, a predominantly negative relation with internalizing symptoms could be demonstrated [17, 35]. Thus, the more internalizing symptoms that children exhibit, the less that they are able to ensure their own rights and desires when interacting with others.

In preschool age, children are faced with various social challenges, characterized by the transition from a familiar environment with primary caretakers to a peer-dominated environment and new caretakers. Therefore, children must develop new self-regulation and social competence skills in this age [37, 38]. Furthermore, mental disorders diagnosed at preschool age often persist or even deteriorate into middle childhood, adolescence and adulthood [39, 40]. Thus, preschool age may be regarded as a sensitive period for both the development of social competence as well as mental health, and therefore it is the age of interest for the current study. Thus, in the current study we focus on the relation between the two dimensions of social competence and externalizing and internalizing symptoms in preschoolers. Recent research has demonstrated robust effects within these relations, mainly ranging from small to medium effect sizes. Nonetheless, it has also raised new questions–for example, in terms of the relation between internalizing symptoms and prosocial behavior or externalizing symptoms and social initiative–that may be addressed by behavioral observation methods (for an overview, see [41]).

### Limitations of previous research

Previous research has shown that social competence may be related to early childhood psychopathological symptoms, although some important limitations appear. First, studies have often focused on questionnaire-based data using teachers’ or parents’ reports to assess children’s social competence [34, 35, 42]. While these measures enable comprehensively and efficiently assessing children’s social competence, this approach also contains methodological problems, whereby teachers and parents only have a limited perspective on children as they see them predominantly in one context (e.g., at school or home). However, the social context strongly influences the expression and thus the observability of children’s behavior, given that a school setting with peers might challenge different competences than a home setting with parents (and maybe siblings). Thus, competence ratings are often variable depending on the social context of the rater [43]. In addition, caregivers’ questionnaire-based ratings may exhibit some bias; for example, due to social desirability, the type of problem to be assessed, or caregivers’ mental constitution [44]. Furthermore, measuring different variables (e.g., symptoms and competence) with the same instrument may incorporate the risk of a *shared method variance* [45, 46]. This means that associations between the two variables could be artificially influenced by a common measurement error or a general answering tendency of the rater. Behavioral observation in a standardized setup–such as is common in developmental research with preschoolers (e.g., [12, 47, 48]) and clinical research with older children exhibiting manifest disorders [49–51]–can complement questionnaire data and provide objective and precise measures of children’s social competence. However, in clinical research with preschoolers, these methods are scarcely used when examining social competence (for an overview, see [41]).

Second, behavioral observation methods may also shed new light on the heterogeneous results of previous research in the context of internalizing symptoms that we assume to be largely driven by informants’ perspective. When integrating children’s own perspective, mainly positive relations between prosocial behavior and internalizing symptoms have been found (e.g. [35, 36]), whereas when the raters were solely caregivers, mainly negative relations between prosocial behavior and internalizing symptoms have been found (e.g. [34, 17]). As internalizing symptoms are more difficult to recognize from the outside than–for example–externalizing symptoms, caregivers tend to underestimate children’s internalizing symptoms [52–54]. Children with internalizing symptoms themselves may exhibit a dysfunctional notion of helping or comforting others; for example, in the context of pathological altruism driven by parentification processes (for an overview, see [55]). Thus, the relationship between prosocial behavior and internalizing symptoms may differ depending on the raters’ perspective.

Third, previous studies have often used global measures of social competence such as the quality of peer relations (e.g., [2,8]) and have rarely investigated the two dimensions of social competence separately. However, this appears inevitable, as previous research has demonstrated that prosocial behavior and social initiative are not only independent dimensions [17] but also appear to be specifically impaired depending on whether the child mainly exhibits externalizing or internalizing symptoms [41]. Thus, when investigating the relationship between social competence and mental health in children, the measurement of social competence should regard the dimensions of prosocial behavior and social initiative separately.

Fourth, a large body of previous work on the relationship between children’s social competence and psychopathology has only assessed children’s symptoms from caregivers’ perspectives. However, since children’s reporting of symptoms has already provided meaningful information by preschool -age (e.g., [56, 57]) and since children may report problems that are not always visible to caregivers [52–54], their perspective should also be taken into account.

### The current study

In the current study, we aimed to investigate whether prosocial behavior and social initiative measured in a standardized behavioral observation task are related to externalizing and internalizing symptoms in preschoolers. For this task, we created an attractive resource limited in time. According to Green and Rechis [6], limited resources provide an important socialization function for preschoolers as they have to negotiate turns with others and manage interpersonal conflicts. Thus, both self- and other-oriented social competence are necessary to successfully manage such situations. Turn-taking is regarded as a human-unique skill that is an inevitable basis to sustain long-term cooperative relationships [16]. In order to obtain an encompassing picture of children’s internalizing and externalizing symptoms, we assessed both caregivers’ (parents’ and teachers’) and children’s perspectives.

Based on previous findings, we hypothesize that externalizing symptoms from caregivers’ and children’s perspectives are negatively related to prosocial behavior and positively related to social initiative in our task [4, 24, 30–32]. We further hypothesized that internalizing symptoms from caregivers’ and children’s perspective are negatively related to social initiative in our task [17, 34]. Due to heterogeneous findings on internalizing symptoms in previous studies [17, 34–36], we hypothesized an association between internalizing symptoms and prosocial behavior but no assumptions about the direction of the effect. Due to differences between informants in previous research [43], we also hypothesize informant-dependent differences but make no assumption about the direction of the effect.

## Material and methods

### Participants and procedure

This study was part of a larger study on social-emotional competence and psychopathological symptoms at preschool age. The participants were 117 children (*M*age = 4 years 8 months, *SD* = 2 months, 23 days, age range = 4 years 5 months to 5 years 3 months, females = 57), who were recruited via local childcare centers. The study strictly adhered to the legal requirements of the country in which it was conducted. All parents gave written informed consent, and ethical approval was granted by the Ethical Committee at the Medical Faculty of the University of Leipzig (No: 358/16-ek). Five children were excluded from analysis due to procedural error (*n* = 2), participant non-compliance with the experimental procedure (*n* = 2), or the incapacity of the child to understand the experimental apparatus (*n* = 1).

The test sessions took place in a separate room in children’s day care centers on two different test days. The behavioral observation task was conducted with 111 children (usually on day 1), while the Berkeley Puppet Interview (BPI; [58]) was conducted with 97 of these children (usually on day 2). The remaining fourteen children were not able to participate in the second test session due to longer vacations, sickness or inattention regarding the BPI. The German version of the Strengths and Difficulties Questionnaire (SDQ-Deu; [59]) was handed to the teacher with the main caretaking assignment, and 109 teachers returned the SDQ. Parents received the SDQ in combination with an addressed and pre-stamped envelope, and 77 sent it back to our department. Data collection was conducted in 25 daycare centers, which were evenly distributed across Leipzig, a medium-sized German city. Thus, socioeconomic status as well as other demographic variables are expected to be evenly distributed in our sample of children, teachers, and parents.

### Behavioral measure of social competence

#### Setup

**Fig 1:**
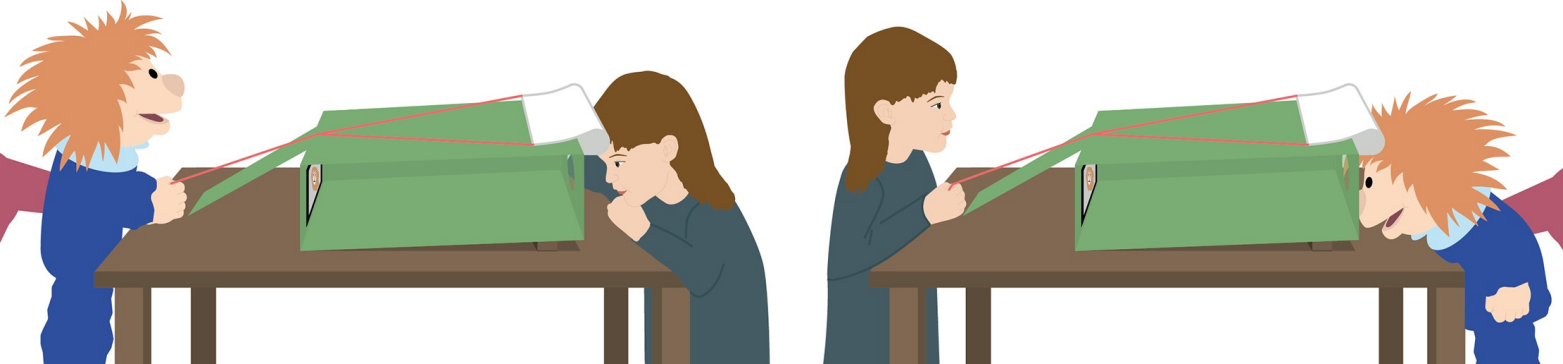
Setup of the movie viewer paradigm. The left panel shows the situation in which the child was in the watching position and prosocial behavior was measured; the right panel shows the situation in which the child was in the helping position and social initiative was measured.

We constructed an attractive resource limited in time for preschool-age children (for an overview of limited resources, see [6]): a *movie viewer*, displayed in Fig 1. This resource was based on previous studies in which the principle of a similar movie viewer proved effective [60, 61]. The movie viewer had a tablet PC inside that showed eight animal movies (lasting 30 s each), accompanied by pleasant music that faded in and out at the beginning and end of a movie. One could look inside the viewer through a peek hole at the front side of the box. However, a curtain covered this peek hole. One person could only look inside the box if another person pulled a string at the backside to lift the curtain. Therefore, cooperation was essential to watch a movie. Children in the present study interacted with a hand puppet called Maxi. We had a female and a male version of Maxi, whose use was counterbalanced over all children. Using a hand puppet ensured highly standardized study procedures. Developmental research has shown that preschool-age children’s motivation and attention may increase when using puppet interactions (see [33]).

#### Design and procedure of the movie viewer

First, there was a warm-up session in which an experimenter acted as the moderator. He introduced the hand puppet and the child to each other and explained them how to use the movie viewer. The second experimenter who acted as the puppeteer hid herself behind the puppet so that the child was exclusively interacting with the puppet. Afterwards, the moderator randomly assigned either the child or the puppet to the watching position, told them that they could take turns, and left the room. The assignment to the watching and helping position was counterbalanced over all children. If the child was in the watching position, we measured prosocial behavior (offering the puppet to watch; see Table 1). If the child was in the helping position, we measured social initiative (asking the puppet for a turn; see Table 1). Thus, all children took part in both positions. If the child did not do or say anything while watching or helping, the puppet gave verbal prompts in a prescribed time sequence to provoke a reaction from the child. In each position, there was a maximum of five prompts within approximately 130 s, which were part of a ramping-up procedure (see also [62]), continuously increasing in their explicitness. For example, if the puppet was in the watching position, the first and thus weakest prompt was “this is a nice movie”, while the fifth and thus strongest prompt was “would you like to watch now?”. If the child showed social initiative after one of these prompts (e.g. stated the wish to switch positions), the puppet gave up the watching position and stopped prompting. If the puppet was in the helping position, the first and thus weakest prompt was “this sounds like a nice movie”, and the fifth and thus strongest prompt was “may I also watch a movie?”. If the child showed prosocial behavior after one of these prompts (e.g. offered the puppet to watch the movies), the puppet gave up the helping position and stopped prompting. Thus, there could be a maximum of five prompts to provoke the relevant turn-taking element in both the prosocial behavior and the social initiative session. As there was no significant effect of trial order, we included prosocial behavior and social initiative as independent predictors in the subsequent linear models.

**Table 1:** Overview of the movie viewer task.

Variable	Operationalization	Coding	Range
Prosocial Behavior	Giving a turn to the puppet in the movie viewer	Time a child needed until giving a turn to the puppet, structured by prompts	0 (fast)– 5 (slow)
Social Initiative	Demanding a turn for oneself in the movie viewer	Time a child needed until demanding a turn for itself, structured by prompts	0 (fast)– 5 (slow)

This procedure was piloted with sixteen children to ensure that children understood the experimental procedure and to adapt the time slots between prompts. Changes within the procedure, verbal instructions, and time slots were continuously made until the setup was satisfactory. A detailed description of the experimental procedure is presented as supporting information to this manuscript (see S1 File).

#### Coding

During the experimental procedure, we measured the time that each child took after a prompt until he/she reacted with a prosocial or social initiative action. Both prosocial behavior and social initiative were coded on a metric scale from 0 to 5. The individual reaction time of a child after a prompt was held in relation to the average time between two prompts. This strategy enabled us to build a metric score between 0 and 5 for each child, which facilitated subsequent data analysis. For example, if children reacted exactly in the middle between the beginning and the first prompt, they received 0.5. Afterwards, the prosocial behavior and social initiative scale were reversed, so that a high score on the scale corresponded to a high level of prosocial behavior/social initiative. Excellent interrater reliability was established for 25% of the sample for prosocial behavior, ICC = 0.99, and for social initiative, ICC = 0.93. For an overview of the operationalization and coding of prosocial behavior and social initiative, see Table 1.

### Psychometric measures of psychopathological symptoms

#### Strengths and Difficulties Questionnaire (SDQ; [63])

Teachers and parents completed the German version of the SDQ (SDQ-Deu; [59]). Five items each measure the severity of conduct problems, hyperactivity, emotional problems, peer problems, and prosocial behavior. As proposed by the original authors [64], these scales were grouped into second-order scales of internalizing symptoms (emotional problems, peer problems) and externalizing symptoms (conduct problems, hyperactivity). Each item in the SDQ is rated on a three-point scale. Internal consistency was highly acceptable for teachers’ ratings of externalizing symptoms (Cronbach’s alpha = .84) and internalizing symptoms (Cronbach’s alpha = .84), and for parents’ ratings of externalizing symptoms (Cronbach’s alpha = .72) and internalizing symptoms (Cronbach’s alpha = .70). Other studies using a multi-informant assessment yielded comparable internal consistencies in the SDQ (e.g. [36]).

#### Berkeley Puppet Interview (BPI; [58])

The BPI is a standardized and validated interview that uses hand puppets to assess children’s own perspective on their health and well-being. Six to seven items each measure the scales of oppositional-defiant behavior, impulsivity/hyperactivity, aggressive behavior, depression, over-anxiety, separation anxiety, prosocial behavior, and social inhibition, which can be grouped into second-order scales of internalizing symptoms (depression, over-anxiety, separation anxiety) and externalizing symptoms (oppositional-defiant behavior, aggressive behavior, hyperactivity/impulsivity). Children were interviewed by two graduate students. During the BPI, two hand puppets made opposing statements on symptoms and competence and asked the children about their position.

Internal consistency was highly acceptable for children’s ratings of externalizing symptoms (Cronbach’s alpha = .72) and internalizing symptoms (Cronbach’s alpha = .73) and was in accordance with the results of other studies that have successfully implemented the BPI [36, 57]. The German version of the BPI has also shown feasible in a clinical sample with five-year old children [56].

### Data analysis strategy

The primary analyses of this study were based on linear models. Separate models for teachers’, parents’ and children’s perspectives were calculated in each of the subsequent steps. Dependent variables were either children’s externalizing symptoms or their internalizing symptoms. Independent control variables were age, gender, and the opposite symptom dimension (externalizing or internalizing symptoms), while the independent variables of interest were children’s prosocial behavior and social initiative assessed in the behavioral observation task. Linear models including bootstrapped coefficients and confidence intervals were calculated using *lavaan* in R. As some assumptions for linear models such as a normal distribution of residuals were violated, we decided to bootstrap our results. An a priori power analysis revealed sufficient statistical power for expected effects based on previous studies in the field (see [41]).

## Results

### Descriptive statistics

Means and standard deviations of symptoms and social competence are shown in Table 2. Descriptive statistics for all relevant subscales and a correlation matrix of all variables of interest are presented as supporting information to this manuscript (see S1 Table, S2 Table and S3 Table). The behavioral observation measure of prosocial behavior was not significantly associated with the SDQ (teachers and parents) and BPI ratings of prosocial behavior.

**Table 2:** Means (*M*) and Standard Deviations (*SD*) of subordinated scales.

	Teachers		Parents		Children	
	*M*	*SD*	*M*	*SD*	*M*	*SD*
Externalizing symptoms	4.87^a^	4.06	5.40^a^	3.18	57.61^b^	13.44
Internalizing symptoms	2.76^a^	3.37	2.69^a^	2.51	63.25^b^	14.34
Prosocial behavior movie viewer	-	-			2.34^c^	1.73
Social initiative movie viewer	-	-			2.31^d^	1.80

^a^ = SDQ scale range: 0–20

^b^ = BPI scale range: 20–140

^c^ = Scale range of prosocial behavior in the behavioral task: 0–4.67

^d^ = Scale range of social initiative in the behavioral task: 0–4.60.

### Prediction of children’s externalizing symptoms

#### Teachers’ perspective

The results of the regression analysis of externalizing symptoms (SDQ ratings) on age, gender, and internalizing symptoms (SDQ ratings), and prosocial behavior (behavioral task) and social initiative (behavioral task) are shown in Table 3. Neither prosocial behavior in the behavioral task nor social initiative were significant predictors of externalizing symptoms. The R^2^ for the model was 0.19.

**Table 3:** Regression analysis of externalizing symptoms (teachers’ perspective, SDQ).

	*B* (β)	*SE B* (β)	*p*	*ci*.*lower*	*ci*.*upper*
Internalizing Symptoms	0.37 (0.31)	0.14 (0.11)	.01	0.13	0.66
Age	0.04 (0.02)	0.14 (0.09)	.80	-0.24	0.30
Gender	-2.05 (-0.26)	0.71 (0.09)	< .01	-3.48	-0.67
Prosocial behavior (behavioral task)	-0.41 (-0.19)	0.32 (0.14)	.20	-1.11	0.21
Social initiative (behavioral task)	0.54 (0.23)	0.34 (0.14)	.11	-0.11	1.23

*B =* unstandardized regression weight, β = standardized regression weight, *SE B* = standard error of B, *p* = probability value, *ci*.*lower* = lower bound of the confidence interval after bootstrapping *B*, *ci*.*upper* = upper bound of the confidence interval after bootstrapping *B*.

#### Parents’ perspective

The results of the regression analysis of externalizing symptoms (SDQ ratings) on age, gender, and internalizing symptoms (SDQ ratings), and prosocial behavior (behavioral task) and social initiative (behavioral task) are shown in Table 4. Neither prosocial behavior in the behavioral task nor social initiative were significant predictors of externalizing symptoms. The R^2^ for the model was 0.22.

**Table 4:** Regression analysis of externalizing symptoms (parents’ perspective, SDQ).

	*B* (β)	*SE B* (β)	*p*	*ci*.*lower*	*ci*.*upper*
Internalizing Symptoms	0.43 (0.34)	0.12 (0.09)	.001	0.20	0.68
Age	-0.30 (-0.27)	0.13 (0.11)	0.02	-0.53	-0.03
Gender	-0.97 (-0.15)	0.67 (0.10)	0.15	-2.29	0.37
Prosocial behavior (behavioral task)	-0.03 (-0.02)	0.28 (0.15)	0.91	-0.62	0.52
Social initiative (behavioral task)	0.37 (0.20)	0.29 (0.15)	0.20	-0.18	1.01

*B =* unstandardized regression weight, β = standardized regression weight, *SE B* = standard error of B, *p* = probability value, *ci*.*lower* = lower bound of the confidence interval after bootstrapping *B*, *ci*.*upper* = upper bound of the confidence interval after bootstrapping *B*.

#### Children’s perspective

The results of the regression analysis of externalizing symptoms (BPI ratings) on age, gender and internalizing symptoms (BPI ratings), and prosocial behavior (behavioral task) and social initiative (behavioral task) are shown in Table 5. Prosocial behavior in the behavioral task was a significant negative predictor of externalizing symptoms, while social initiative was no significant predictor. The confidence interval of prosocial behavior does not include zero, and thus the effect is regarded as robust. The R^2^ for the model was 0.27. The reported effect remained significant when including SDQ ratings of prosocial behavior by parents and teachers in the models. This extra analysis provides evidence of a specific predictive value of behavioral observation data additionally to common questionnaire measures.

**Table 5:** Regression analysis of externalizing symptoms (children’s perspective, BPI).

	*B* (β)	*SE B* (β)	*p*	*ci*.*lower*	*ci*.*upper*
Internalizing symptoms	0.44 (0.47)	0.08 (0.08)	< .001	0.28	0.60
Age	-0.37 (-0.08)	0.53 (0.11)	0.48	-1.34	0.64
Gender	-5.94 (-0.22)	2.46 (0.08)	0.02	-10.82	-1.25
Prosocial behavior (behavioral task)	-1.92 (-0.26)	0.79 (0.10)	0.02	-3.38	-0.29
Social initiative (behavioral task)	1.34 (0.17)	0.79 (0.11)	0.09	-0.38	2.85

*B =* unstandardized regression weight, β = standardized regression weight, *SE B* = standard error of B, *p* = probability value, *ci*.*lower* = lower bound of the confidence interval after bootstrapping *B*, *ci*.*upper* = upper bound of the confidence interval after bootstrapping *B*.

### Prediction of children’s internalizing symptoms

#### Teachers’ perspective

The results of the regression analysis of internalizing symptoms (SDQ rating) on age, gender and externalizing symptoms (SDQ ratings), and prosocial behavior (behavioral task) and social initiative (behavioral task) are shown in Table 6. Neither prosocial behavior in the behavioral task nor social initiative were significant predictors of internalizing symptoms. The R^2^ for the model was 0.14.

**Table 6:** Regression analysis of internalizing symptoms (teachers’ perspective, SDQ).

	*B* (β)	*SE B* (β)	*p*	*ci*.*lower*	*ci*.*upper*
Externalizing symptoms	0.28 (0.33)	0.08 (0.10)	<0.001	0.13	0.44
Age	-0.10 (-0.08)	0.16 (0.13)	0.56	-0.43	0.20
Gender	-0.21 (-0.03)	0.64 (0.10)	0.75	-1.53	1.07
Prosocial behavior (behavioral task)	0.33 (0.18)	0.26 (0.14)	0.20	-0.23	0.79
Social initiative (behavioral task)	-0.42 (-0.22)	0.29 (0.16)	0.15	-0.94	0.20

*B =* unstandardized regression weight, β = standardized regression weight, *SE B* = standard error of B, *p* = probability value, *ci*.*lower* = lower bound of the confidence interval after bootstrapping *B*, *ci*.*upper* = upper bound of the confidence interval after bootstrapping *B*.

#### Parents’ perspective

The results of the regression analysis of internalizing symptoms (SDQ rating) on age, gender and externalizing symptoms (SDQ ratings), and prosocial behavior (behavioral task) and social initiative (behavioral task) are shown in Table 7. Social initiative in the behavioral task was a significant negative predictor of internalizing symptoms, while prosocial behavior was no significant predictor. Nonetheless, the confidence interval of social initiative nearly includes zero, and thus interpretation is restricted and to be treated with caution. The R^2^ for the model was 0.16.

**Table 7:** Regression analysis of internalizing symptoms (parents’ perspective, SDQ).

	*B* (β)	*SE B* (β)	*p*	*ci*.*lower*	*ci*.*upper*
Externalizing symptoms	0.28 (0.36)	0.10 (0.10)	<0.01	0.11	0.48
Age	0.10 (0.11)	0.09 (0.10)	0.29	-0.08	0.28
Gender	-0.01 (-0.01)	0.60 (0.12)	0.98	-1.17	1.16
Prosocial behavior (behavioral task)	0.16 (0.11)	0.21 (0.15)	0.45	-0.26	0.56
Social initiative (behavioral task)	-0.48 (-0.32)	0.23 (0.14)	0.04	-0.92	-0.03

*B =* unstandardized regression weight, β = standardized regression weight, *SE B* = standard error of B, *p* = probability value, *ci*.*lower* = lower bound of the confidence interval after bootstrapping *B*, *ci*.*upper* = upper bound of the confidence interval after bootstrapping *B*.

#### Children’s perspective

The results of the regression analysis of internalizing symptoms (BPI ratings) on age, gender and externalizing symptoms (BPI ratings), prosocial behavior (BPI ratings) and prosocial behavior and social initiative (behavioral task) are shown in Table 8. Prosocial behavior in the behavioral task was a marginally significant positive predictor of internalizing symptoms, while social initiative was no significant predictor. Nonetheless, the confidence interval of prosocial behavior includes 0, and thus interpretation is restricted and to be treated with caution. The R^2^ for the model was 0.27.

**Table 8:** Regression analysis of internalizing symptoms (children’s perspective, BPI).

	*B (β)*	*SE B (β)*	*p*	*ci*.*lower*	*ci*.*upper*
Externalizing symptoms	0.50 (0.47)	0.10 (0.08)	< .001	0.32	0.71
Age	0.69 (0.14)	0.55 (0.11)	0.21	-0.42	1.69
Gender	2.58 (0.09)	2.57 (0.09)	0.32	-2.35	7.59
Prosocial behavior (behavioral task)	1.95 (0.25)	1.03 (0.90)	0.06	-0.01	3.95
Social initiative (behavioral task)	-0.38 (-0.05)	1.09 (0.13)	0.73	-2.64	1.80

*B =* unstandardized regression weight, β = standardized regression weight, *SE B* = standard error of B, *p* = probability value, *ci*.*lower* = lower bound of the confidence interval after bootstrapping *B*, *ci*.*upper* = upper bound of the confidence interval after bootstrapping *B*.

## Discussion

The present study aimed to investigate the relationship between two facets of social competence–prosocial behavior and social initiative–and teachers’, parents’ and children’s assessments of psychopathological symptoms in a preschool-age community sample. The assessment of social competence via standardized behavioral observation extends previous research, which has mostly focused on questionnaire-based caregiver assessments (for an overview, see [41]). Hereby, we provided an objective and precise measure of social competence that is less vulnerable to informants’ bias than in questionnaire-based assessments, for example. Behavioral observation data of less prosocial behavior proved to be a meaningful predictor for children’s ratings of greater externalizing symptoms, while greater prosocial behavior was a marginally relevant predictor for children’s ratings of greater internalizing symptoms. Behavioral observation data of less social initiative proved to be a meaningful predictor for parents’ ratings of greater internalizing symptoms. The strongest and most robust effect was the negative relation between prosocial behavior in the behavioral observation and children’s ratings of externalizing symptoms.

### Externalizing symptoms

When predicting externalizing symptoms from teachers’, parents’ and children’s perspectives separately, regression analysis showed a significant contribution of our behavioral observation data on social competence only for children’s perspective. In accordance with our hypotheses, externalizing symptoms rated by children were negatively related to prosocial behavior. Thus, the higher the ratings on externalizing symptoms given by children, the less prosocial behavior that children showed in the behavioral task. Accordingly, the more externalizing symptoms that children exhibited, the less often they offered a turn in the movie viewer to the puppet and the more that they demanded a turn in the movie viewer for themselves. This effect remained significant when teachers’ and parents’ ratings of prosocial behavior in the SDQ were also included in the models showing a specific predictive value of behavioral observation data on children’s externalizing symptoms additionally to common questionnaire measures. This finding indicating deficient other-oriented social behavior in children with early externalizing symptoms is in accordance with previous questionnaire-based studies [4, 30–32], but it also extends previous research demonstrating the effect in a behavioral observation paradigm that is less sensitive to biases than questionnaire-based approaches. It also extends previous research demonstrating this finding within children’s ratings of externalizing symptoms. Contrary to previous studies (e.g. [24, 36]), we did not find externalizing symptoms to be related to reduced prosocial behavior in teachers’ and parents’ perspectives. It seems that this specific form of prosocial behavior is more strongly reflected within children’s ratings. It could be that teachers and parents rather report a broader picture regarding children’s competence that is not reflected in the concrete test situation. The non-existent finding of social initiative and externalizing symptoms is consistent with previous research that has also not found this relation in questionnaire-based assessment [4, 42], even though aggressive behavior may be related to assertiveness and social participation at kindergarten age (see [4]).

Thus, via standardized behavioral observation, we demonstrated specific effects of other-oriented social competence on preschoolers’ ratings of externalizing symptoms, partly supporting the prediction in the tripartite model of social competence of Perren and Malti [21].

### Internalizing symptoms

When predicting internalizing symptoms from teachers’, parents’ and children’s perspectives, regression analysis showed a significant contribution of our behavioral observation data of social competence. In line with our hypotheses, we found that internalizing symptoms rated by parents were negatively related to social initiative, while internalizing symptoms rated by children were positively related to prosocial behavior. Thus, the higher the ratings on internalizing symptoms given by parents, the less social initiative that children showed in the movie viewer, while the higher the ratings on internalizing symptoms given by children, the more prosocial behavior that children showed in the movie viewer. This means that the more internalizing symptoms that children exhibited, the more they offered a turn in the movie viewer to the puppet and the less they demanded a turn in the movie viewer for themselves. However, these effects did not appear quite robust and have to be treated with caution.

The finding that children’s ratings of internalizing symptoms were positively related to prosocial behavior in the behavioral task corresponds to the heterogeneous results in previous research including studies mainly finding a positive relation [35, 36] when children’s perspectives were included, and mainly finding a negative relation when caregivers’ perspectives were included [17, 34]. Thus, the behavior measured in the movie viewer seems to more strongly correspond to children’s internal states than caregivers’ perceptions. Positive relations between prosocial behavior and internalizing symptoms can also be interpreted in the context of pathological altruism [65], which describes an excessive orientation towards others’ needs that are evident–for example–in excessive prosocial behavior. As Zahn-Waxler and colleagues [55] point out, both very low and very high levels of prosocial behavior can be a risk factor in children’s development. Future studies should therefore also consider quadratic relations between the prosocial behavior and psychopathological symptoms, especially internalizing symptoms. Thus, it could be the case that different perspectives also tap different difficulties: caregivers’ ratings might better identify deficient behavioral expressions of prosocial behavior, while children ratings might better identify dysfunctional beliefs. The behavioral observation data in our study more strongly corresponded to children’s internal states or personal behavioral conduct. However, this interpretation also has to be treated with caution as the underlying effect did not appear to be particularly robust.

### Summary

In sum, we found that a behavioral observation of prosocial and social initiative is meaningful when assessing preschool-age psychopathology, although it largely depends on the perspective on symptoms. Behavioral observation allows for precise operationalization of social competences, which facilitates the assessment of specific strengths and difficulties in these children. We demonstrated that externalizing symptoms in children’s ratings were accompanied by deficient prosocial behavior and internalizing symptoms in children’s ratings were accompanied by excessive prosocial behavior. For parents’ ratings of internalizing symptoms, the behavioral observation data on social competence also yielded meaningful results. We found that internalizing symptoms were accompanied by deficient social initiative in the behavioral task. Teachers’ ratings of externalizing and internalizing symptoms were not predictable by neither prosocial behavior nor social initiative in the behavioral observation task. Teachers mainly observe children in larger groups in which many different actions occur at the same time. One possible explanation for the absent teacher effects could be that teacher are “better raters” for a rather broad assessment of social competence, whereas the specific forms of behavior such as in the behavioral observation task are not reflected in their ratings of externalizing symptoms.

### Limitations

There are also several limitations to the current study. In our behavioral observation task, we measured quite circumscribed forms of social competence in one specific situation. The behavioral observation measure of prosocial behavior was not associated with SDQ and BPI scales of prosocial behavior. As Dunfield [66] delineates, different facets of prosocial behavior such as helping, sharing and comforting behavior are independent dimensions varying in the onset and course of development. Thus, on the one hand, our measure benefits from the absent correlation to SDQ and BPI measures: compared with the rather broad assessment in the SDQ and BPI that incorporates various facets of prosocial behavior, we measured a specific form of prosocial behavior in a concrete situation. Therefore, our measure can be regarded as innovative and was still related to pathology variables. On the other hand, it would be important to investigate whether our results are replicable in other studies investigating a similar sample of children. It would further be important to demonstrate that our results are transferable to other forms of psychopathology (e.g. in older children or in children with clinically-revenant disorders) and prosocial behavior (e.g. sharing, comforting, see [66]). Furthermore, subjective experiences of social situations are not measurable with behavioral observations. Therefore, it would be important to differentiate even more between the differential predictive value of both children subjective experiences *and* their observable behavior. Moreover, despite no evidence in our study that children did not accept the puppet as an interaction partner, it would be a meaningful extension to include real peers as an interaction partner. Future studies could implement this point; for example, by having two trained children (one female and one male) who act as the interaction partner for all children who are tested. Thus, individual characteristics of the interaction partner would be controlled for, while ecological validity would be enhanced.

As our design is cross-sectional, it was not possible to determine causal risk factors based on our results. In order to investigate causal pathways concerning the influence of social competence in a behavioral task on psychopathological symptoms, longitudinal studies are necessary. Longitudinal effects of behavioral observation data on social competence on psychopathological symptoms would also provide greater benefits to these methods for diagnostics and interventions in childhood psychopathology. Furthermore, because our results are based on a community sample, implications for prevention and intervention are only suggestive and need to be refined by further studies including a clinical sample. Additionally, we were not able to assess further demographic information on children, teachers, and parents due to privacy restrictions regarding data collection in children’s daycare centers. Especially for the comparison of different groups of children (e.g. control vs. clinical), future research should control for confounding factors. Nonetheless, we are confident with the distribution of teacher, parent and child variables in our sample as we visited 25 different kindergartens throughout all city parts, including areas with higher and lower average socioeconomic status.

### Conclusion and practical implications

Our study holds some important implications for theory and practice. Externalizing symptoms were found to be accompanied by deficient prosocial behavior in the behavioral task, emphasizing the need for interventions to focus on fostering other-oriented forms of social behavior. In contrast to externalizing symptoms, internalizing symptoms were found to be accompanied by excessive prosocial behavior and deficient social initiative in the behavioral task. This finding emphasizes the importance of fostering self-oriented social behavior and reducing augmented care for others while disregarding one’s own needs. In sum, behavioral methods are applicable to the assessment of interpersonal deficits associated with externalizing and internalizing symptoms. Findings based on methods such as the paradigm used in the current study have to be replicated and transferred to other samples. Consequently, in the long run, behavioral observation methods are a promising extension of existing questionnaires and interviews for future research, diagnostics and interventions at preschool age.

## Supporting information

S1 Table Means (*M*) and Standard Deviations (*SD*) of children’s symptoms and prosocial behavior from teachers’ and parents’ perspective (SDQ Ratings). SDQ scale range: 0–10.
(DOCX)

S2 Table Means (*M*) and Standard Deviations (*SD*) of children’s symptoms and prosocial behavior from children’s perspective (BPI Ratings). ^a^ = BPI scale range: 7–49, ^b^ = BPI scale range: 6–42.
(DOCX)

S3 Table Spearman correlation coefficients between teachers’, parents’, and children’s assessments of symptoms and prosocial behavior and the behavioral observation of social competence. Probability-values in parentheses.
(DOCX)

S1 File Movie viewer procedure. (DOCX)
