# Supplementary material for: Behavioral observation of prosocial behavior and social initiative is related to preschoolers’ psychopathological symptoms
Source: PLoS One. 2019 Nov 21;14(11):e0225274. doi: 10.1371/journal.pone.0225274 (PMC6874079; doi:10.1371/journal.pone.0225274)
Supplement: S1 Table — SDQ scale range: 0–10. (DOCX) [file pone.0225274.s001.docx]

**S1 Table. Means (*M*) and Standard Deviations (*SD*) of Children’s Symptoms and Prosocial Behavior from Teachers’ and Parents’ Perspective (SDQ Ratings).** SDQ scale range: 0-10.

|  | Teachers | | Parents | |
| --- | --- | --- | --- | --- |
|  | *M* | *SD* | *M* | *SD* |
| Conduct problems | 1.54 | 1.78 | 1.83 | 1.51 |
| Hyperactivity | 3.34 | 2.80 | 3.57 | 2.24 |
| Peer problems | 1.28 | 1.90 | 1.00 | 1.18 |
| Emotional problems | 1.48 | 1.97 | 1.69 | 1.76 |
| Prosocial behavior | 7.29 | 2.17 | 7.82 | 1.67 |
