# Supplementary material for: Behavioral observation of prosocial behavior and social initiative is related to preschoolers’ psychopathological symptoms
Source: PLoS One. 2019 Nov 21;14(11):e0225274. doi: 10.1371/journal.pone.0225274 (PMC6874079; doi:10.1371/journal.pone.0225274)
Supplement: S2 Table — a = BPI scale range: 7–49, b = BPI scale range: 6–42. (DOCX) [file pone.0225274.s002.docx]

**S2 Table. Means (*M*) and Standard Deviations (*SD*) of Children’s Symptoms and Prosocial Behavior from Children’s Perspective (BPI Ratings).** ^a^ = BPI scale range: 7-49, ^b^ = BPI scale range: 6-42.

|  | *M* | *SD* |
| --- | --- | --- |
| Depression | 20.14^a^ | 6.05 |
| Separation anxiety | 21.34^b^ | 7.09 |
| Over-anxiety | 22.77^a^ | 6.43 |
| Oppositional defiant behavior | 17.92^b^ | 5.49 |
| Inattention/hyperactivity | 23.87^a^ | 8.65 |
| Aggressive behavior | 16.81^a^ | 4.62 |
| Prosocial behavior | 33.71^a^ | 7.84 |
