# Supplementary material for: Behavioral observation of prosocial behavior and social initiative is related to preschoolers’ psychopathological symptoms
Source: PLoS One. 2019 Nov 21;14(11):e0225274. doi: 10.1371/journal.pone.0225274 (PMC6874079; doi:10.1371/journal.pone.0225274)
Supplement: S3 Table — Probability-values in parentheses. (DOCX) [file pone.0225274.s003.docx]

**S3 Table. Spearman Correlation Coefficients between Teachers’, Parents’, and Children’s Assessments of Symptoms and Prosocial Behavior and the Behavioral Observation of Social Competence.** Probability-values in parentheses.

|  | a | b | c | d | e | f | g | h | i | j | k |
| --- | --- | --- | --- | --- | --- | --- | --- | --- | --- | --- | --- |
| *Teachers (SDQ)* |  |  |  |  |  |  |  |  |  |  |  |
| a Externalizing symptoms | 1 |  |  |  |  |  |  |  |  |  |  |
| b Internalizing symptoms | 0.36 (<.001) | 1 |  |  |  |  |  |  |  |  |  |
| c Prosocial behavior | -0.72 (<.001) | -0.29 (<.01) | 1 |  |  |  |  |  |  |  |  |
| *Parents (SDQ)* |  |  |  |  |  |  |  |  |  |  |  |
| d Externalizing symptoms | 0.26 (.02) | 0.19 (.09) | -0.21 (0.07) | 1 |  |  |  |  |  |  |  |
| e Internalizing symptoms | -0.05 (.68) | 0.42 (<.001) | -0.01 (.90) | 0.32 (<.01) | 1 |  |  |  |  |  |  |
| f Prosocial behavior | -0.20 (.09) | -0.03 (0.80) | 0.19 (0.10) | -0.49 (<.001) | -0.12 (.30) | 1 |  |  |  |  |  |
| *Children (BPI)* |  |  |  |  |  |  |  |  |  |  |  |
| g Externalizing symptoms | 0.18 (.09) | -0.07 (.50) | -0.31 (.002) | 0.18 (.13) | -0.24 (.04) | -0.07 (.55) | 1 |  |  |  |  |
| h Internalizing symptoms | 0.03 (.79) | 0.09 (.37) | 0.01 (.97) | -0.01 (0.92) | 0.01 (.98) | 0.06 (.59) | 0.44 (<.001) | 1 |  |  |  |
| i Prosocial behavior | -0.09 (.37) | 0.09 (0.37) | 0.20 (0.05) | -0.09 (.46) | 0.02 (.88) | 0.19 (.10) | 0.06 (0.53) | 0.24 (0.02) | 1 |  |  |
| *Behavioral observation (movie viewer)* |  |  |  |  |  |  |  |  |  |  |  |
| j Prosocial behavior | 0.09 (.38) | -0.02 (.86) | -0.07 (.47) | 0.11 (.36) | 0.01 (.91) | -0.03 (.82) | -0.10 (0.33) | 0.14 (0.19) | -0.06 (0.52) | 1 |  |
| k Social initiative | 0.12 (.20) | -0.20 (.04) | -0.08 (.39) | 0.06 (.59) | -0.20 (.08) | 0.10 (.41) | 0.06 (.57) | 0.18 (.07) | 0.02 (.85) | 0.65 (<.001) | 1 |
