# Supplementary material for: Behavioral observation of prosocial behavior and social initiative is related to preschoolers’ psychopathological symptoms
Source: PLoS One. 2019 Nov 21;14(11):e0225274. doi: 10.1371/journal.pone.0225274 (PMC6874079; doi:10.1371/journal.pone.0225274)
Supplement: S1 File — (DOCX) [file pone.0225274.s004.docx]

**S1 file. Movie viewer procedure**

Setting: There is a colored Box on the table (movie viewer). The front side of the viewer is covered by a curtain which can be lifted by a string. The string runs from the curtain at the front side of the viewer to the back side of the viewer where one can pull it in order to lift the curtain. Once lifted, it is possible to look inside the viewer at the front side through two peek holes. The inside of the viewer contains a tablet computer which shows animal movies. These movies can be started and stopped by a remote control. In total, there are 8 movies lasting 30 s each, accompanied by pleasant music. Between two movies, a black screen appears that fades out at the beginning of a movie and fades in at the end of a movie, thus serving as a turn-taking cure.

Experimenter 1 (E1) instructs the game, experimenter 2 (E2) plays the puppet called “Maxi” with her left hand and operates the movie viewer via remote control with her right hand.

**I Introduction**

***E1*** *picks up the child from the kindergarten group. Smalltalk on the way to the test room.*

**E1:** „I will show you something special here in this room. Come on, let’s have a look at it.”

***E1*** *and the child enter the test room in which E2 is already hidden beyond the puppet Maxi awaiting E2 and the child.*

**E1:** „So, [name of the child], I would like to introduce someone to you. This is Maxi, he/she [counterbalanced] would like to play with you today.”

**Maxi:** „Hello [name of the child]. My name is Maxi. Nice to see you! I am excited about playing with you today.”

*Smalltalk between Maxi und the child to become acquainted. Topics: age, favorite food, person who usually picks them up from kindergarten.*

**E1:** „Now, I will show you what I brought here for you. Look (*points to the viewer*), this is a movie viewer.”

**Maxi**: „Oh, great!”

**E1**: „In this movie viewer, you can watch wonderful animal movies”.

**Maxi:** „Oh, that sounds nice. I really want to watch these movies”.

*Break for reactions of the child.*

**E1:** „But there is a very important point. (*short break*) Only one of you at a time can look into the movie viewer. The other one stands at the back side of the table and pulls this string *(points to the string)* in order to lift the curtain at the front side of the viewer. Only then, the one in the watching position may look inside and watch a movie. I will show to you how that works!

***E1*** *approaches* *the other side of the table and pulls the string so that the curtain is lifting.*

**II Game**

**E1:** „So, please come to me both of you. *(Maxi and the child go to E1)*. Now… *(short break)*. I have forgotten to manage something outside. But you two can stay here and play with the movie viewer.

1. „Maxi, you may start watching the movies. You can take turns, if you want.” ***E1*** *leaves the room.*

**Maxi**: „Oh, great!”

**Session 1: Maxi watches**

*Maxi watches the first movie.*

**Maxi**: “I see animals, that is great”. *E2 stops during the break after the 1^st^ movie.*

*Waiting time (silence for 5 seconds = 1^st^ prompt): Does the child demand a turn for himself/herself? level 1*

- *If the child demands a turn, go on to session 2.*
- *If not, Maxi watches another movie.*

**Maxi**: “I see animals, that is great”.

*E2 stops during the break after the 2^nd^ movie.*

**Maxi** (2^nd^ prompt): „That was an exciting movie.“ *(silence for 5 seconds)* level 2

- *If the child demands a turn, go on to session 2.*
- *If not:*

**Maxi** (3^rd^ prompt): „You would have liked it as well.“ *(silence for 5 seconds)* level 3

- *If the child demands a turn, go on to session 2*
- *If not, Maxi watches another movie.*

**Maxi**: „I see animals, that is great”.

*E2 stops during the break after the 3^rd^ movie.*

**Maxi** (4^th^ prompt): „That was an exciting movie.“ *(silence for 5 seconds)* level 4

- *If the child demands a turn, go on to session 2.*
- *If not:*

**Maxi** (5^th^ prompt): „Would you also like to watch a movie?” *(silence for 5 seconds)* level 5

- *If the child demands a turn, go on to session 2*
- *If not, Maxi says:* “Now I see a black screen. I will leave the watching position now and you can watch a movie.” *Go on to session 2*

**Session 2: Child watches**

*The child and Maxi change positions. The child watches a movie. E2 stops during the break after the 1^st^ movie. Maxi does not say anything.*

*Waiting time (silence for 5 seconds = 1^st^ prompt): Does the child offer a turn to Maxi? level 1*

- *If the child offers a turn, go on to session 3.*
- *If not, the child watches another movie.*

*E2 stops during the break after the 2^nd^ movie.*

*If the child does not say anything:*

**Maxi** (2^nd^ prompt): „It seems that this was an exciting movie.“ *(silence for 5 seconds)* level 2

- *If the child offers a turn to Maxi, go on to session 3.*
- *If not:*

**Maxi** (3^rd^ prompt): „I think I would have liked it as well.“ *(silence for 5 seconds)* level 3

- *If the child offers a turn to Maxi, go on to session 3.*
- *If not, the child watches another movie.*

*E2 stops during the break after the 3^rd^ movie.*

*If the child does not say anything:*

**Maxi** (4^th^ prompt): „It seems that this was an exciting movie.“ *(silence for 5 seconds)* level 4

- *If the child offers a turn to Maxi, go on to session 3.*
- *If not:*

**Maxi** (5^th^ prompt): „Can I also watch a movie?” *(silence for 5 seconds)* level 5

- *If the child offers a turn, go on to session 3.*
- *If not, Maxi says:* “I will leave the helping position now and you can pull again the string.” *Go on to session 3.*

**Session 3: Maxi the child watch**

*No coding anymore. Maxi and the child may watch until the movies end.*

***E1*** *returns.* „Hello again. Have you played with the movie viewer?“ *(leave time to answer)*.

**Maxi**: „ Yes, it was great. I saw many beautiful fishes. It was a lot of fun playing with you [name of the child]. Now I am a bit tired. So, I say goodbye and hope to play with you again some time. Bye bye!“.

*Maxi leaves the room. E1 hands the postcard to the child, the two leave the room and E1 brings the child back to his/her kindergarten group.*

1. Counterbalancing: The child starts.

**E1**: „[Name of the child], you may start watching the movies. You can take turns, if you want.” ***E1*** *leaves the room.*

*Similar procedure as in version a) but flipped (Session 1: the child watches, session 2: Maxi watches, Session 3: Maxi and the child watch).*

**Notes**

- Version a) and version b) counterbalanced.
- If the child asks questions during Maxi’s watching or helping time in session 1 or session 2, Maxi remains silent. If the child specifically wants to know anything about the content of the movies, Maxi only reacts saying “ohhh”.
- If the child turns away from the watching or helping position without saying anything, Maxi asks: “and now?”. If the child does not respond after two subsequent queries from Maxi, Maxi leaves its position and asks: “shall we take turns now?”
- Maxi does not lower the curtain in the helping position as long as the child does not react.
